# Methicillin-resistant Staphylococcus aureus as a cause of chronic wound infections: Alternative strategies for management

**DOI:** 10.3934/microbiol.2022011

**Published:** 2022-04-24

**Authors:** Oriana Simonetti, Samuele Marasca, Matteo Candelora, Giulio Rizzetto, Giulia Radi, Elisa Molinelli, Lucia Brescini, Oscar Cirioni, Annamaria Offidani

**Affiliations:** 1 Clinic of Dermatology, Department of Clinical and Molecular Sciences, Polytechnic University of Marche, Ancona, Italy; 2 Clinic of Infectious Diseases, Department of Biomedical Sciences and Public Health, Polytechnic University of Marche, Ancona, Italy

**Keywords:** MRSA, biofilm, quorum sensing, wound infection, antibiotic resistance

## Abstract

Biofilm formation at the level of a wound plays an important role in its chronicization. The difficulty of its eradication has driven research toward the discovery and synthesis of new molecules that can act on biofilm to promote wound healing. This narrative review focuses on alternative molecules that can act and promote the eradication of methicillin-resistant *Staphylococcus aureus*, taking into consideration its antibiotic resistance, virulence, tendency toward the tenacious colonization of wounds by biofilms, and its increased prevalence in both community and hospital settings. A selection of promising studies were reported, analyzing the in vitro and/or in vivo efficacy of bacteriophages, metal nanoparticles, RNAIII inhibiting peptide (RIP), synthetized RIP derivatives, proteinase K and hamamelitannin.

## 1. Introduction

Methicillin-resistant *Staphylococcus aureus* (MRSA) is a current problem not only in the hospital but also in community settings. Although, in some countries, the incidence of hospital-acquired (HA)-MRSA is under control, due to the active surveillance and antibiogram-based therapy, community-associated MRSA (CA-MRSA) is found more and more frequently. MRSA often shows resistance against almost every beta-lactams and other antimicrobial agents [1],[2].

A distinction should be made between HA-MRSA and CA-MRSA in regard to therapeutic targets, because, in an environment like a hospital, HA-MRSA can acquire a lot of other mechanisms of defense against different classes of antibiotics, whereas CA-MRSA is still susceptible to different classic antimicrobial drugs [1],[2].

In particular, the ability of staphylococci to produce biofilm, i.e., a strong protective factor against antibiotics and bactericidal molecules, plays a very important role. Bacteria organized in biofilms, compared to planktonic bacteria, have a superior resistance to antibiotics by several orders of magnitude [3]–[5]

Biofilm formation is characterized by an initial phase of adhesion to organic or inorganic surfaces, followed by proliferation, exodus, maturation with the formation of microcolonies and, finally, a dispersal phase, all within a three-dimensional extracellular matrix, where the various bacteria communicate closely with each other [6]. This extracellular matrix, which is composed of exopolysaccharides, proteins and DNA molecules, [5] creates a suitable environment for inter-bacterial communication, information exchange and increased resistance to external agents of various natures (i.e., physical, chemical and biological) [5]. Biofilm also plays a very important role in chronic ulcers. Wound chronicity is a global and widespread problem. The prevalence of individuals with long-term sequelae caused solely by pressure ulcers was estimated to be approximately one million in 2015 [7]. The prevalence of chronic ulcers worldwide appears to be approximately 2.21 per 1000 people within a population. More specifically, the prevalence for chronic lower extremity ulcers, i.e., the most frequent type with a venous-related cause, is 1.51 per 1000 inhabitants [8]. In addition to the main underlying causes, such as reduced venous return, inadequate arterial supply, or alterations in wound healing due to impaired glucose metabolism, colonization and, in some cases, subsequent bacterial infection, more often polymicrobial and multidrug-resistant, delay the healing of these wounds [9]–[12]. Once the integrity of the skin barrier is lost, the subepidermal tissues come into contact with the external environment, losing sterility. Bacterial colonization is inevitable; however, when the wound tends to persist, the possibility of bacteria organizing into more complex structures, giving rise to the biofilm, is increased. This may further reduce the possibility of wound healing due not only to a physical barrier action, but also to a prolonged persistence of the inflammatory phase [13],[14]. The different evolutionary phases of the biofilm, from formation to dispersion, as well as the production of virulence factors, are mediated by inter-bacterial communication that allows regulated and harmonized gene expression. This is enabled by small molecules called autoinducers that, being mediated by bacterial density, induce signal transduction mechanisms that will result in a modification of gene expression once they reach a certain concentration [9],[15].

## 2. Materials and methods

We performed a narrative review of the literature by searching PubMed for the following keywords alone or in combination: quorum-sensing, MRSA, wound infection, antibiotic resistance and biofilm. Only studies in English were included. The purpose was to provide a review of current or future strategies to target MRSA quorum sensing and biofilm formation. A selection of the available literature was made to highlight some of the most promising therapies that have been proven in vivo or have shown efficacy in vitro. We decided not to limit the time frame of the articles since some old articles could actually have been a starting point for new research areas. This narrative review is intended to be our opinion on old and new technologies under development that may be used in the near future in clinical practice.

## 3. Results

### 3.1. Bacteriophage therapy

Bacteriophages, also called phages, are viruses that target specific strains and species of bacteria for replication [16]. Bacteriophages appear to play a role in MRSA quorum sensing and biofilm production. Some phages, including ones infecting vibrio cholerae or bacillus subtilis, appear to be able to exploit quorum-sensing signals to guide the choice between the lytic and lysinogenic cycles, and to enter the lytic cycle [17],[18]. From a therapeutic point of view, the preferred phages are lytic, because they cause bacterial lysis and do not integrate into the host cell DNA, unlike temperate phages [19]. Phage infection appears to be impaired by biofilm; however, some of these viruses developed counter mechanisms, such as depolymerase synthesis, which can weaken the biofilm matrix and bacterial capsule, promoting contact with receptors on the bacterial membrane [20]. Łusiak-Szelachowska et al. observed that bacteriophages, single or in cocktails, or alone or in combination with classical antibiotics, as well as lysine derived from phages, can disrupt the biofilm in addition to yielding direct bactericidal action [21].

The use of phages in combination with each other or with other treatments, or, alternatively, the use of engineered phages in the fight against biofilm-forming bacterial infections, is essential to increase their efficacy and reduce the risk of resistance development, even against these viral weapons [19],[22].

In vitro, *ex vivo* and *in vivo* studies have shown excellent synergy in regard to the combination of phages and antibiotics against biofilm in urinary tract infections, odontogenic infections and infections following prosthetic implants, even against MRSA [21].

In a case report Doub et al. describe the ability to eradicate a chronic prosthetic MRSA infection using a pool of lytic bacteriophage, previously investigated microbiologically to assess its efficacy, administered both intra-articular and intravenous, in combination with daptomycin. Previous eradication attempts failed with vancomycin, daptomycin and doxycycline [23].

Jikia et al., in a case report, demonstrated how two patients with post-radiation skin lesions and MRSA infections had poor and slow improvement despite the application of topical and systemic antibiotics. However, following the application of PhagoBioDerm, a biodegradable polymeric matrix containing multiple phage species with different targets, the purulent exudate was significantly reduced within 2 days, and complete eradication of MRSA was observed within 7 days [24]. Markoishvili et al. tested PhagoBioDerm on 107 patients with chronic ulcers (predominantly venous stasis ulcers) who were previously treated with various topical and systemic drugs with little benefit. Following topical phage therapy, alone or combination with other treatments, healing was observed in 70% of the 96 patients who completed the follow-up. Complete eradication of microorganisms was confirmed in 22 of the subjects by performing microbiological analysis [25]. The five subjects (5%) who did not respond to treatment had diabetic ulcers [25]. Positive results for diabetic ulcers were observed in vivo in animal models. Chhibber et al. compared the efficacies of a treatment with linezolid, phage MR-10, no treatment, or a combination of both treatments on a distal lesion in diabetic mice [19]. Although both treatments as a monotherapy yielded overlapping results in terms of microbial load and eradication timelines, the combination treatment showed greater efficacy from Day 1, significantly reducing the bacterial load isolated from the wound [26]. However, a mouse model is not always representative of what could be expected in human in vivo studies.

### 3.2. Metal nanoparticles

Abd El-Hamid et al. evaluated the biofilm-inhibiting and antimicrobial action of zinc oxide and silver nanoparticles. The anti-biofilm activity seemed to be due to the inhibition of two genes: *icaA* and *agr* [27]. In addition, metal nanoparticles have different mechanisms of antimicrobial action, i.e., binding at the bacterial membrane to destroy the lipid and protein molecules, and the production of reactive oxygen species mediated by zinc (Zn^++^) and silver (Ag^+^) cations [28]. Several other cationic nanoparticles seem to possess the same biofilm inhibition ability, and be mediated by the same mechanisms of action [29].

Ansari et al. isolated different strains of *Staphylococcus epidermidis* and *S. aureus* with methicillin-resistant patterns from skin lesions like purulent dermatitis and burns; they observed antimicrobial action with minimum inhibitory concentration (MIC), ranging from 11.25 to 45 µg/mL. Moreover, evaluating the anti-biofilm action of AgNPs in vitro on Congo red agar plates, they observed that even low concentrations of 10 µg/mL caused a block in the production of the glycocalyx matrix, thus inhibiting the formation of the biofilm. Increasing the AgNP concentration causes the anti-biofilm activity to increase, until an arrest of microbial growth occurs at high concentrations [30].

In vivo studies on animals and patients with skin lesions were carried out using nanoparticles with different cations that were also conjugated with different substances, demonstrating in each case faster wound healing than comparator groups [31]–[33]. Specifically, in the case of an infected wound, faster mouse wound healing was observed clinically and histologically following the use of nanoparticles, in comparison to the absence of treatment, the use of antibiotics such as gentamicin [32] or the use of substances based exclusively on hyaluronic acid [34]. As a tool to minimize the risk of systemic toxicity by reducing the amount of silver particles while maintaining the same antibacterial efficacy, hydrogels seem to be very promising [35]. Haidari et al. demonstrated the efficacy of ultra-small AgNP hydrogel against *S. aureus* biofilm in vitro and in vivo using a murine model. They also observed safety and biocompatibility in vitro, and the absence of signs of organ damage in vivo in mice [36].

### 3.3. RNAIII inhibiting peptide

RNAIII inhibitor peptides (RIPs) are heptapeptides that compete with the RNAIII activating protein (RAP) molecule to inhibit the phosphorylation of targets of the RNAIII activating protein (TRAPs), which are responsible for the regulation of toxins, the accessory gene regulator (*agr*) system, adhesion proteins and other molecules involved in the virulence of *S. aureus* strains [37]–[40].

Several studies conducted in vitro and in animal models have shown that the use of RIPs as a quorum-sensing inhibitor, used both prophylactically and therapeutically, results in significant reductions of MRSA colonization and infection on different medical devices, including catheters and prostheses; it also results in faster wound healing. This efficacy, which is comparable or greater than that of teicoplanin, is significantly increased when they are used in combination with other classical antibiotics, showing very high synergism [41]–[44].

It is important to point out that, in in-vitro studies, the MIC and minimum bactericidal concentration (MBC) are not modified when evaluated on bacteria in planktonic culture rather than on biofilms; this is reasonable given the mechanism of action [41]–[44].

RIPs have also been used in some case studies on skin lesions, including difficult wounds such as ulcers on diabetic feet. It was observed that, following combination therapy, chronic ulcers tended to heal much faster, behaving similarly to acute wounds [45].

### 3.4. RIP derivatives synthetized

Numerous molecules have been synthesized from RIPs via single amino acid substitution or amino acid removal; the purpose was to investigate whether these new peptides also had the ability to inhibit quorum sensing, and whether this ability was greater than that of the original molecule. Among these molecules, we recognize FS8, FS3 and FS10.

A study has shown that the FS8 molecule, derived from RIPs with substitution of phenylalanine in Position 7 with alanine, has prophylactic efficacy against MRSA biofilm in in vitro and in vivo cases of mouse models. Efficacy was also observed when it was applied in combination with tigecycline; this yielded a synergistic effect. Simonetti et al. demonstrated intraperitoneal tigecycline antibiotic therapy; a vascular graft bonded with the FS8 was placed in a subcutaneous pouch and applied before MRSA injection [46]. The same results were obtained in a similar study wherein the molecules investigated were FS3 and daptomycin. FS3 is characterized by the substitution of a serine molecule by alanine at Position 2. Again, the single preventive treatments resulted in a lower microbial load, whereas the combination of the two showed a synergistic effect [47].

In contrast to the first two molecules, FS10 was evaluated in mouse models because of its therapeutic action on skin and subcutaneous MRSA infection. FS10 is a tetrapeptide, which differs from RIPs because it lacks the first and last two amino acids of the chain. A study [48] previously compared the infected control group to a group using antibiotic-free dressings, a group treated with intraperitoneal tigecyclin, a group treated with an FS10-soaked Allevyn dressing and a group treated with the FS10-soaked dressing and tigecycline. The best preventative effect, both microbiologically and histologically, was observed for combination therapy [48]. In addition, all three molecules showed a greater capacity for quorum-sensing inhibition than the original molecule [46]–[48].

### 3.5. Proteinase K

Numerous studies in vitro show that *S. Aureus* surface proteins play a role in the development of infections and biofilms [27],[49]. Among them, biofilm-associated protein (Bap) plays a major role in biofilm formation and early adhesion. The enzymatic proteinase K is able to impact the biofilm development of several isolated *S. aureus* strains. It has exhibted an increased ability to disperse the biofilm of Bap-positive *S. aureus*; however, it had no effect on the biofilm of Bap-negative strains. Proteinase K also appears to be able to result in the down-regulation of the expression of the *agaA* and *agr* alleles implicated in biofilm formation and quorum sensing. Moreover, the combined use of topical and systemic antibiotics and proteinase K showed that the latter is able to increase the efficacy of antibiotics themselves. In particular, proteinase K is able to make a larger area of biofilm susceptible to the action of the antibiotic. In fact, the biofilm forms a physical barrier that prevents the spread of the antibiotic. Proteinase K allows the antibiotic to penetrate deeper into the biofilm and kill bacterial cells. Bap has four binding sites for the Ca^2+^ ion, so it was evaluated whether binding to this ion was capable of inhibiting biofilm degradation by proteinase K. The results showed that there was no significant difference in biofilm growth in the presence of proteinase K alone or with increasing levels of Ca^2+^. In other words, there was no difference in proteinase K action for the biofilm samples with or without Ca^2+^. Enzymatic degradation of *S. Aureus*-induced biofilm is emerging as a novel strategy to fight resistant infections related to the biofilm itself [50].

### 3.6. Hamamelitannin

Hamamelitannin (HAM) is a natural polyphenol belonging to the tannin family. HAM is able to inhibit RNAIII production, bacterial colonization and the virulence of *S. aureus*. In particular, HAM is able to interfere with the RAP/TRAP quorum-sensing system. RAP/TRAP is one of the two quorum-sensing systems by which *S. aureus* regulates its virulence together with the agr system; both of them can modify gene expression through the control of RNAIII. HAM also acts on the agr system. However, this does not appear to be its main mechanism. It does not seem to have a direct effect on bacterial growth even when used at high concentrations, so it cannot be considered a traditional antibiotic. HAM also seems to cause an increase in the efficacy of antibiotics. Specifically, the combined use of HAM and vancomycin has indicated superior efficacy in terms of eliminating *S. aureus* Mu50 biofilm in an *in vitro* experiment (as compared to vancomycin alone) [27],[51]. The mechanism by which HAM increases the efficacy of vancomycin is complex. HAM leads to a reduction in the expression of several genes involved in the biosynthesis of peptidoglycan precursors, such as *glmS*, *lysC*, *asd* and *dapA*, by decreasing cell wall thickness. In addition, HAM results in the reduction of eDNA concentration in the biofilm matrix, thereby increasing its susceptibility. The reduction in cell wall thickness and the reduction in eDNA concentration cause an increase in *S. aureus* susceptibility to the antibiotic. HAM appears to increase the efficacy of not just vancomycin, but also of other antibiotics, through cell wall changes and eDNA reduction; this includes beta lactams and daptomycin. An increase in the elimination of cells belonging to the *S. aureus* biofilm has been reported when antibiotics such as cephalozine, cephalonium, linezolid, tobramycin, fusidic acid, cephalexin or cefoxitin have been used in combination with HAM. HAM could also be useful in preventing biofilm formation on medical devices [27],[51],[52].

Cobrado et al. observed *in vivo* efficacy in murine models; biofilm formation by *S. aureus*, *S. epidermidis* and *Acineotbacter baumannii* was prevented, demonstrating HAM superiority over cerium nitrate and chitosan [53]. In similar experiments with murine models and nematodes, the effectiveness against MRSA was also observed. [52],[54]. Kirdan et al. observed that HAM facilitated the healing of a dog's skin infection after antibiotic failure with cefazolin and clavamox [55]. Several other hamamelitannin analogs have been synthesized and have demonstrated efficacy against MRSA biofilm formation in vitro and in vivo [56]–[58].

### 3.7. Ambuic acid

Ambuic acid is a highly functionalized cyclohexenone that is isolated from the endophytic fungus *Pestalotiopsis sp*. It is able to inhibit the "signaling" of quorum-sensing systems in several pathogenic bacteria, including *S. aureus*. In particular, ambuic acid acted on the agr system of quorum-sensing systems at the transcriptional level in vitro and in vivo in mouse models. The specific target seems to be the autoinducing peptide, which activates a signal transduction cascade with the production of RNAII and RNAIII. It appears to be able to prevent tissue injury by MRSA in skin wounding mouse models. Its efficacy is not limited to *S. aureus*, as it also affects other gram-positive bacteria such as *L. monicytogenes* and *E. faecalis*. Moreover, its action seems to be targeted toward pathogenic bacteria, and does not significantly affect the remaining microbial flora [59].

## 4. Discussion

Among the agents most frequently involved in the colonization of skin wounds, we find *S. aureus* [11],[12]. The eradication of biofilms, and/or the prevention of their formation, play an important role in the prevention and treatment of infections resulting from the colonization of devices and skin wounds [13],[41]. In addition, numerous studies have shown that the eradication of the bacterial biofilm promotes and accelerates wound healing, in some cases transforming the behavior of a chronic ulcer into that of an acute wound. The evidence of the important role played by the biofilm has led to the search for molecules that act selectively or predominantly on the biofilm; as this will promote the action of classical antibiotics that, when faced with non-planktonic bacteria, present higher MICs and MBCs [41]. Research has also been carried out on canonical antibiotic molecules to evaluate if some of them also positively affect wound healing. [60]–[62]. In addition to molecules, physical methods such as photodynamics [63] and biological agents such as bacteriophages have also demonstrated a promising effect on wound healing.

Biofilm inhibitors, in most cases, primarily act against biofilm formation or influence quorum sensing, rather than showing a bactericidal action. They act on the biofilm, but not on the planktonic cells; furthermore, even if they exhibit direct antimicrobial activity, its strength is not equal to that of antibiotics; this explains why they often have a weaker effect than antibiotic therapy. However, it has been observed that antibiotics often fail to eradicate the bacteria in the presence of biofilms, and thus result in chronic recurrent infections [41],[11]. Therefore, combination therapy seems to be the best choice. Further studies should be promoted to evaluate combinations of these alternative drugs, in particular bacteriophages, RIPs, or other molecules with quorum quencher action. The field is moving toward the creation of stimuli-responsive treatments/dressings; the use of nanotechnology is being explored to improve the delivery and stability of antimicrobial treatments while reducing their side effects of toxicity. An ideal treatment should provide multifunctional properties, such as effective antimicrobial action, the ability to overcome resistance mechanisms, anti-biofilm action and the ability to promote optimal skin healing [64].

In our opinion, the molecules most likely to find their way into the healthcare device market in the near future are nanoparticle dressings. Metal cations are already in use in humans worldwide, and new research may focus on the use of new vehicles, such as those based in hydrogel, or on the search for cations with higher efficacy or different structures (including ultra-small nanoparticles) that also keep costs down. Bacteriophages have been minimally used in clinical practice, but seem to be well tolerated; they also have selectivity against different bacterial species, as they allow more targeted action against pathogenic microorganisms. One potential problem with this approach is its relatively high cost compared to that of other dressings; however, a theoretical study has indicated a significant reduction in future production costs [65],[66]. RIP and RIP derivatives may be an interesting topical therapy to combine with systemic therapy, considering that there are also human cases. The limitations are the low availability of these molecules and their high costs. There is also a lack of adequate efficacy and safety evaluations considering the small number of patients treated.

## 5. Conclusions

All of these substances seem to be promising as tools to eradicate biofilms and infections and accelerate and/or allow wound healing, especially when combined with more classical antibiotic therapies. Other studies, including case control studies and clinical trials, should be performed to evaluate the efficacy of these alternative treatments. Given the risk of resistance development that is often observed following monotherapies, it is advisable to use combined therapies that act through different mechanisms of action.
